# Association of age at menarche with valvular heart disease: An analysis based on electronic health record (CREAT2109)

**DOI:** 10.3389/fcvm.2023.1029456

**Published:** 2023-04-17

**Authors:** Zhiyu Sun, Yongjie Zhu, Xiaoyan Sun, Zhexun Lian, Mengqi Guo, Xiaohong Lu, Ting Song, Luxin Feng, Yi Zhang, Yawei Xu, Hongwei Ji, Junjie Guo

**Affiliations:** ^1^ Department of Cardiology, The Affiliated Hospital of Qingdao University, Qingdao, China; ^2^ Chinese Patient-Oriented Metabolic and Ischemic Risk Evaluation (CREAT) Study, Qingdao, China; ^3^ Qingdao University, Qingdao Medical College, Qingdao, China; ^4^ Department of Emergency Medicine, The Affiliated Hospital of Qingdao University, Qingdao, China; ^5^ Department of Geriatrics, Qingdao Eighth People's Hospital, Qingdao, China; ^6^ Department of Cardiology, Shanghai Tenth People's Hospital, Tongji University, Shanghai, China; ^7^ Qingdao Municipal Key Laboratory of Hypertension (Key Laboratory of Cardiovascular Medicine), Qingdao, China

**Keywords:** age at menarche, reproductive history, heart valve diseases, electronic health records, risk factors

## Abstract

**Background:**

The association between age at menarche and coronary heart disease has been reported, but the association between age at menarche and valvular heart disease (VHD) has not been described. We aimed to examine the association between age at menarche and VHD.

**Methods:**

By collecting data from four medical centers of the Affiliated Hospital of Qingdao University (QUAH) from January 1, 2016, to December 31, 2020, we sampled 105,707 inpatients. The main outcome of this study was newly diagnosed VHD, which was diagnosed based on ICD-10 coding, and the exposure factor was age at menarche, which was accessed through the electronic health records. We used logistic regression model to investigate the association between age at menarche and VHD.

**Results:**

In this sample (mean age 55.31 ± 13.63 years), the mean age at menarche was 15. Compared with women with age at menarche 14–15 years, the odds ratio of VHD in women with age at menarche ≤13, 16–17, and ≥18 years was 0.68 (95% CI 0.57–0.81), 1.22 (95% CI 1.08–1.38), and 1.31 (95% CI 1.13–1.52), respectively (*P* for all < 0.001). By restricting cubic splines, we found that later menarche was associated with increased odds of VHD (*P* < 0.001). Furthermore, in subgroup analysis of different etiologies, the similar trend persisted for non-rheumatic VHD.

**Conclusions:**

In this large inpatient sample, later menarche was associated with higher risk of VHD.

## Introduction

With advancing age, the prevalence of valvular heart disease (VHD) increased and has become a public health issue (1–4). In 2019, approximately 40.5 million people had rheumatic heart valve disease and 24.2 million people had degenerative mitral valve disease (5). Over the past few decades, despite the reduction of cardiovascular diseases (CVD) in both women and men, it has been increasingly noted that there were significant sex disparities in cardiovascular diseases including VHD (3, 6–8). For example, the prevalence and calcification of aortic stenosis in women were lower than men, and rheumatic lesions were more common in women with mitral regurgitation (9). Sex-specific risk factors may play a role in the development of such sex differences (8).

Age at menarche was one of the early-life reproductive factors in women and was associated with atherosclerotic cardiovascular disease (ASCVD) (10–12). A large cohort study from the United Kingdom found a *U*-shaped association between age at menarche and coronary heart disease (CHD), demonstrating that both early and later menarche were associated with CHD (11). However, the relationship between age at menarche and VHD was unclear, and no research by far had investigated the association between these two. In this study, we sought to examine the association between age at menarche and VHD in a large electronic health record (EHR) database.

## Methods

### Study population

The Chinese patient-oriented metabolic and ischemic risk evaluation (CREAT) study is a retrospective observational study, based on four EHR databases in four medical centers of the Affiliated Hospital of Qingdao University (QUAH) from January 1, 2016, to December 31, 2020 (ChiCTR2100052332). The study protocol was approved by the Institutional Review Board of the Affiliated Hospital of Qingdao University. Due to the deidentification process of the databases, written informed consent was waived. Deidentified data were analyzed in accordance with regulation with approvals from ethics committees.

### Study design, exposure, and outcomes

**Figure 1:**
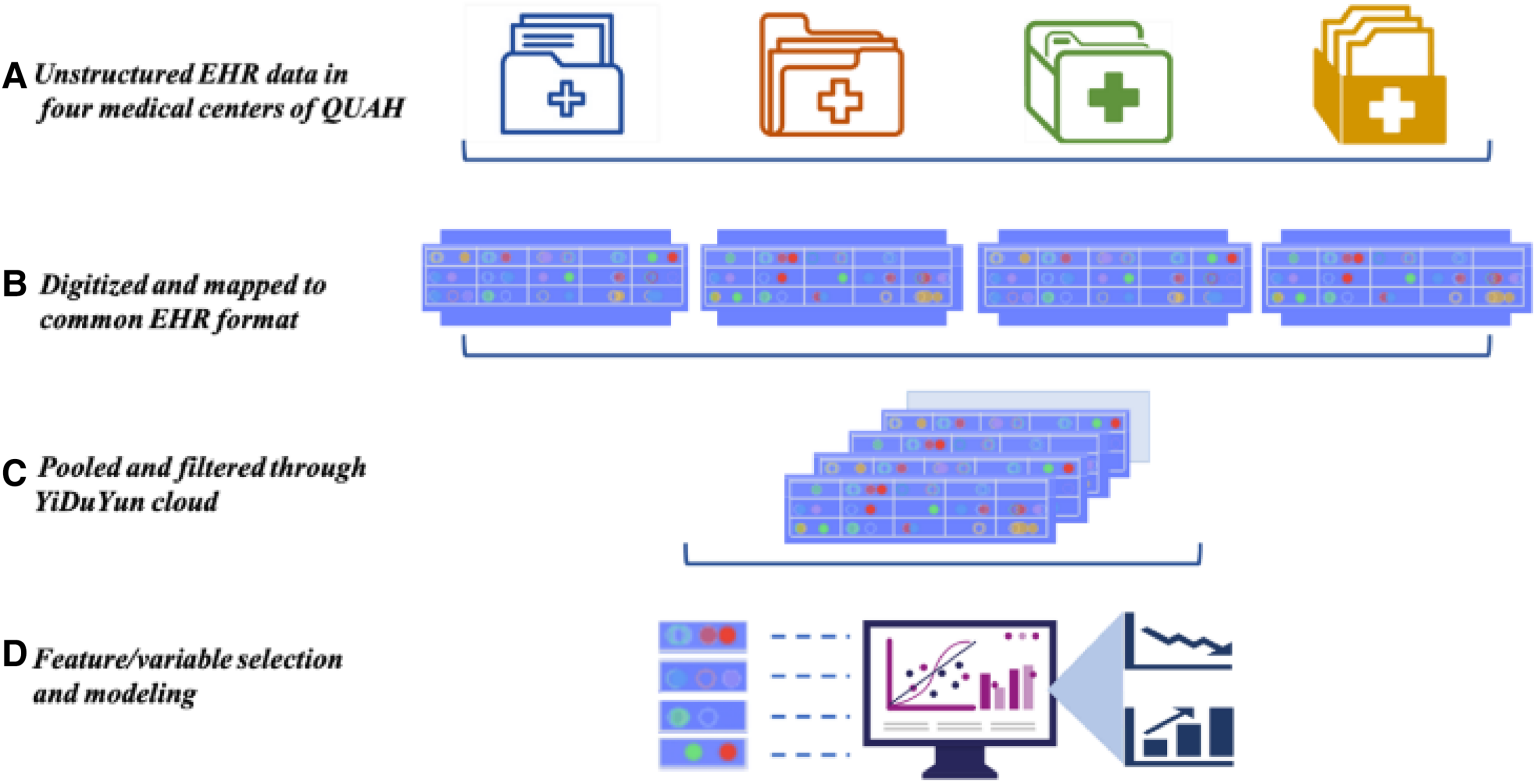
EHR from four medical centers of QUAH. (**A)** EHR data collected from four medical centers. (**B**) Digitization of the collected data and using the standard EHR format. (**C**) Integration and filtering of selected patients. (**D**) Select relevant variables for download and analysis. EHR, electronic health records; QUAH, Qingdao University affiliated hospital.

**Figure 2:**
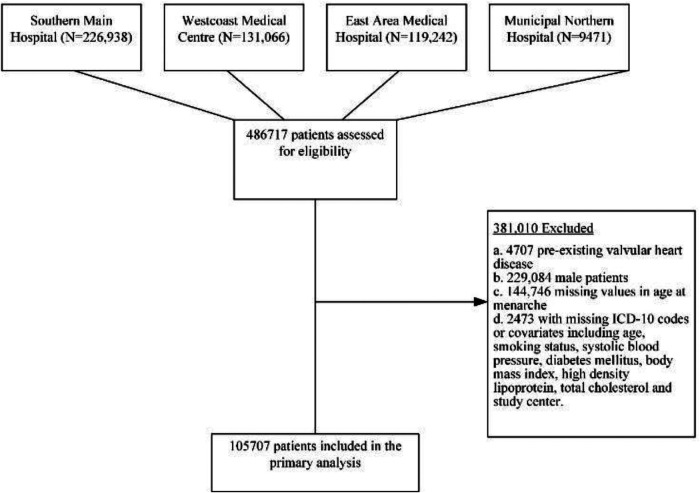
Sampling strategy.

We collected data of 486,717 inpatients through EHR from four medical centers of QUAH (Figure 1). The following demographic and clinical characteristics were obtained from the EHR system: age at menarche; age at admission; sex; smoking status; and blood pressure, lipid profile, body mass index (BMI), and comorbidities as documented by ICD-10 coding. We excluded patients who had missing data on age at menarche or other covariates. We also excluded preexisting VHD cases and only included newly diagnosed VHD patients due to several reasons: (1) some VHDs were congenital heart defects that occurred before menarche, which may lead to difficulty in interpretation; (2) some of the preexisting VHD cases were based on patients’ recalling but not on objectively assessed echocardiography, which was biased; (3) patients with preexisting VHD tended to have longer duration of VHD, and this may lead to survival bias as healthier VHD patients tended to live longer. Among 4,707 self-reported preexisting VHD patients who were excluded, 3,489 had missing data on age at menarche and 1,218 had age at menarche in the EHR. We used chart review to further improve the reliability of diagnosis. We finally included 105,707 qualified inpatients. The details of the sample strategy are shown in Figure 2. To show the robustness of our results, we included preexisting VHD patients with age at menarche in sensitivity analysis. The exposure in this study was age at menarche. According to the distribution of age at menarche, patients were categorized into four groups: ≤13, 14–15 (reference), 16–17, and ≥18 years. The outcome of the study was VHD (i.e., case group). We defined VHD based on International Classification of Diseases, Tenth Revision, Clinical Modification (ICD-10-CM): I05.| I06.| I07.| I08.| I09.1| I09.8| I34.| I35.| I36.| I37.| I38.| I39.| Z95.2| Z95.3| Z95.4.

### Statistical analysis

Continuous variables were expressed as mean (SD) and categorical variables were presented as count (%). The baseline characteristics of the study population were compared using the *t*-test for continuous variables and the χ^2^ test for categorical variables. Multivariable-adjusted logistic regression model was used to examine the association between age at menarche and VHD. Patients who had age at menarche of 14–15 years were used as reference. We fitted a logistic regression model adjusting for covariates including age, smoking status, systolic blood pressure, antihypertensive agents, diabetes mellitus, body mass index, high density lipoprotein cholesterol, and total cholesterol. We also used restricted cubic spline to capture the potential nonlinear association between age at menarche and VHD. Then, we conducted a subgroup analysis by etiology and type of heart valve. For all the models, *P* values were two-sided and considered significant at 0.05. All analyses were performed using R, version 3.5.1 (R Foundation for Statistical Computing).

## Results

A total of 105,707 participants were enrolled in the study. Characteristics of participants are shown in Table 1. The mean age at menarche for women in this cohort was 15 years. A total of 1,668 patients had VHD, and 104,039 patients were considered controls. The mean age of patients with VHD was 64.2 ± 11.0 years, and 58.8% of them took antihypertensive drugs. We observed significant differences between VHD and non-VHD inpatients in BMI, systolic pressure, and lipid profile.

**Table 1:** Characteristics of study participants.

	Non-VHD	VHD	*P*-value
***N***	104,039	1668	—
Non-rheumatic VHD, *n* (%)	—	1,283 (76.9)	—
Age, mean (SD), years	55.17 (13.62)	64.20 (11.00)	<0.001
Smoking, *n* (%)	1,542 (1.5)	56 (3.4)	<0.001
Systolic BP, mean (SD), mmHg	123.23 (18.10)	118.98 (16.92)	<0.001
Antihypertensives, *n* (%)	27,853 (26.8)	980 (58.8)	<0.001
DM, *n* (%)	8,505 (8.2)	186 (11.2)	<0.001
BMI, mean (SD), kg/m^2^	24.55 (3.65)	24.32 (3.92)	0.015
High density lipoprotein cholesterol, mean (SD), mmol/L	1.45 (0.37)	1.23 (0.33)	<0.001
Low density lipoprotein cholesterol, mean (SD), mmol/L	2.86 (0.91)	2.46 (0.89)	<0.001
Total cholesterol, mean (SD), mmol/L	4.63 (1.57)	3.88 (1.55)	<0.001

VHD, valvular heart disease; SD, standard deviation; BP, blood pressure; DM, diabetes mellitus; BMI, body mass index.

**Figure 3:**
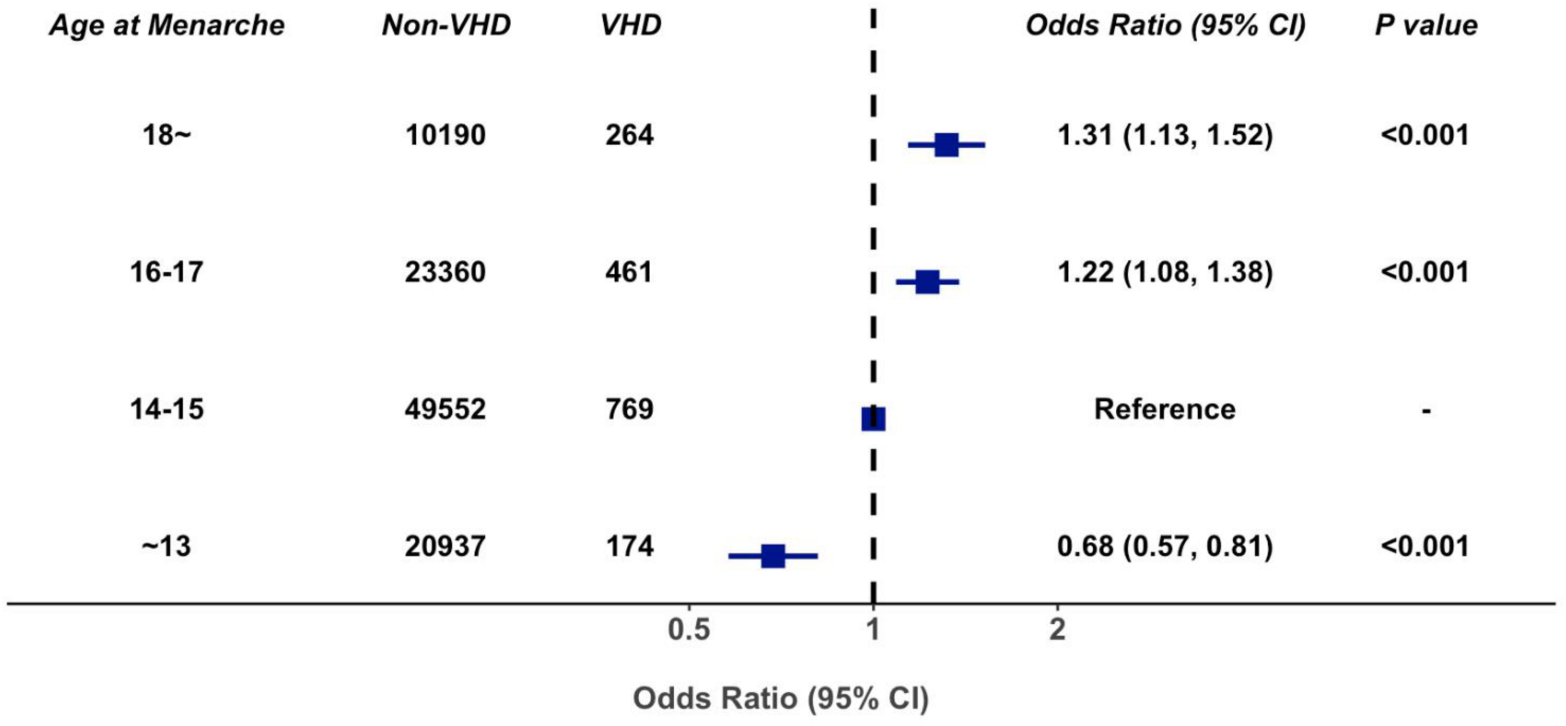
Association of age at menarche with valvular heart disease, adjusted for age, smoking status, systolic blood pressure, antihypertensive agents, diabetes mellitus, body mass index, high density lipoprotein cholesterol, and total cholesterol. 95% CI, 95% confidence interval; VHD, valvular heart disease.

**Figure 4:**
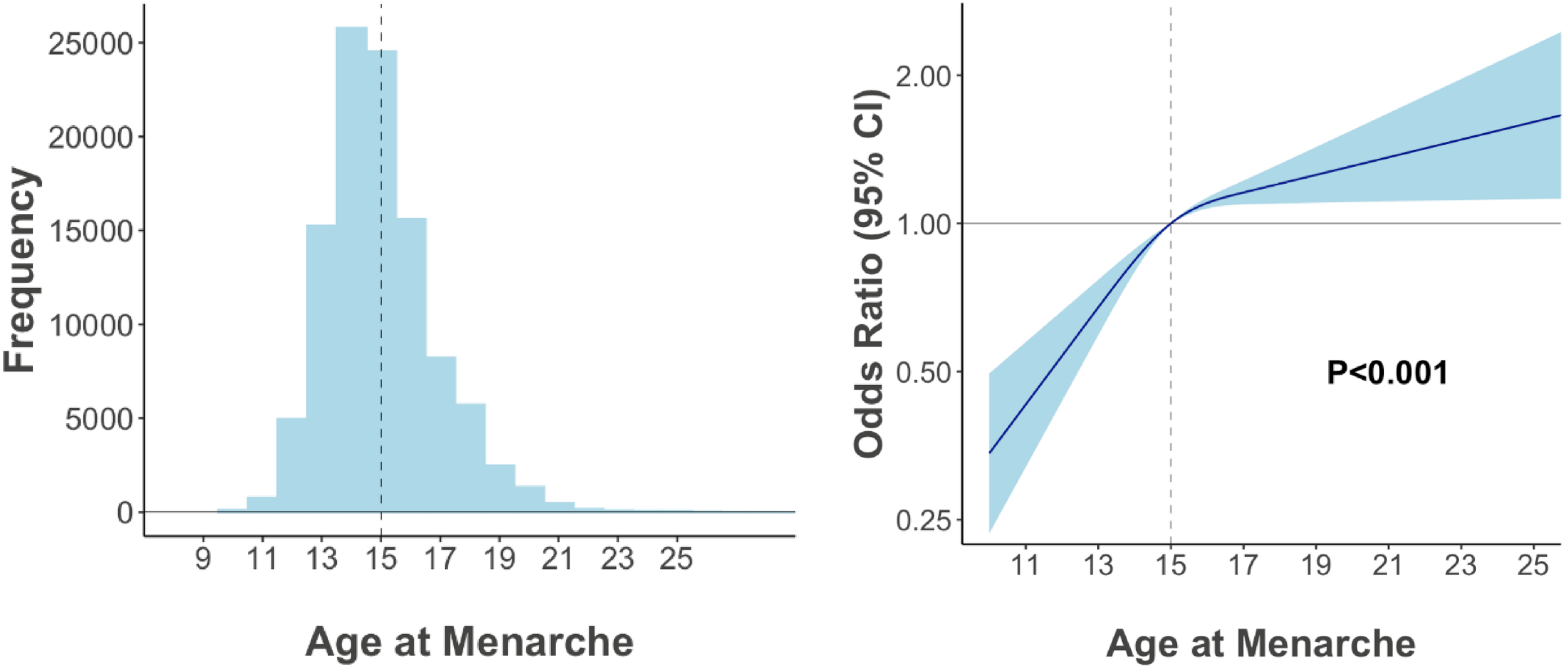
Nonlinear modeling of association between age at menarche and valvular heart disease. Age at menarche was expanded using restricted cubic spline.

**Figure 5:**
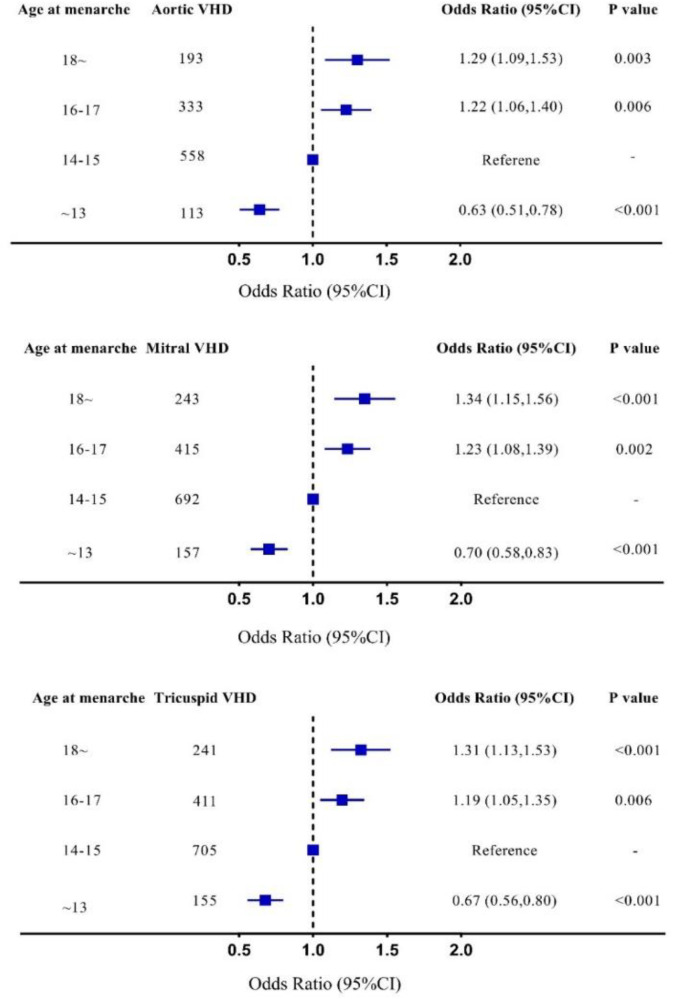
Subgroup analysis in different valves, adjusted for age, smoking status, systolic blood pressure, antihypertensive agents, diabetes mellitus, body mass index, high density lipoprotein cholesterol, and total cholesterol. 95% CI, 95% confidence interval; Mitral VHD, valvular heart disease presents as mitral valve disease; Aortic VHD, valvular heart disease presents as aortic valve disease; Tricuspid VHD, valvular heart disease presents as tricuspid valve disease.

In multivariable-adjusted logistic regression model, compared with patients with age at menarche of 14–15 years, the odds ratio of VHD for age at menarche ≤13, 16–17, and ≥18 years are 0.68 (95% CI 0.57–0.81), 1.22 (95% CI 1.08–1.38), and 1.31 (95% CI 1.13–1.52), respectively (Figure 3). We observed a similar trend when repeating our analysis using restricted cubic spline that later menarche was associated with greater risk of VHD (*P* for trend < 0.001, Figure 4). In subgroup analysis by etiology, we also observed similar association in non-rheumatic VHD (*P* from 0.002 to <0.001). For rheumatic VHD, early menarche (<13 years) was associated with lower risk of VHD (*P* = 0.002), and the odds ratio for later menarche (≥16 years) was 1.2, suggesting that there is no significant violation in directionality, though it is not statistically significant (Table 2). In addition, we analyzed different heart valve types of VHD. Later age at menarche was associated with higher risk of VHD for mitral valve, aortic valve, and tricuspid valve (Figure 5).

**Table 2:** Analysis of different etiological subgroups.

Age at menarche	Non-rheumatic VHD				Rheumatic VHD			
	Case	Control	OR (95% CI)	*P*-value	Case	Control	OR (95% CI)	*P*-value
≥18	209	10,190	1.34 (1.14–1.57)	<0.001	55	10,190	1.28 (0.94–1.74)	0.119
16–17	355	23,360	1.23 (1.08–1.41)	0.002	106	23,360	1.21 (0.95–1.54)	0.124
14–15	583	49,552	Reference	—	186	49,552	Reference	—
≤13	136	20,937	0.72 (0.60–0.87)	0.001	38	20,937	0.58 (0.41–0.82)	0.002

VHD, valvular heart disease; OR, odds ratio; CI, confidence interval.

## Discussion

In this large case–control study consisting of 1,668 VHD cases and more than 100,000 controls, later menarche was associated with increased risk of VHD even after accounting for age, blood pressure, and other risk factors. To the best of our knowledge, this appears to be the first study to examine the association between age at menarche and VHD. In addition, we found that this association was highly consistent across valves in non-rheumatic VHD.

Prior studies in terms of early-life reproductive factors mainly focused on non-valvular ASCVD including CHD (11, 13). Early menarche was reported to be a risk factor for CHD, with age at menarche ≤10 years correlating with higher risk of CHD (14). Meanwhile, the association between age at menarche and ASCVD was also reported to be nonlinear. Lakshman et al. found that early or too late menarche increased the risk of CHD by a large UK cohort study (11); similar observations were also found in other studies (13, 15). Moreover, the association between age at menarche and cardiovascular risk factors was also investigated (16). Lakshman et al. found that early menarche was related to hypertension, type 2 diabetes mellitus, metabolic syndrome, and BMI (12, 17–20). Such cardiometabolic disorders early in life may, in turn, have important implications for vascular health in adulthood.

To date, studies investigating the association between age at menarche and VHD remain scarce. Some studies reported the association between other reproductive factors and VHD. Menopause was the symbol of the end of reproductive life, and its association with VHD has been reported. Honigberg et al. found that natural premature menopause was related to aortic stenosis, and surgical premature menopause was related to mitral regurgitation (21); they also found that hypertensive disorder of pregnancy (HDP) was associated with increased risk of aortic stenosis and mitral regurgitation (22). As another adverse outcome of pregnancy, placental abruption would increase the risk of non-rheumatic VHD (23). Our study expanded prior findings and showed that later menarche was associated with greater risk of VHD. More importantly, in subgroup analysis by different etiologies, we found that etiology had certain impact on the association between age at menarche and VHD. In non-rheumatic VHD, age at menarche was significantly associated with VHD; but in rheumatic VHD, this association was somewhat attenuated. The type of heart valve had no significant effect on this association, and we observed similar findings for mitral valve, aortic valve, and tricuspid valve.

Mechanisms of age at menarche correlating with VHD were complex and largely unknown. This association may be due to the following reasons. The impact of traditional cardiovascular risk factors on cardiovascular health can be broad, which could increase not only the risk of ASCVD but also the risk of VHD. Studies found that later menarche increased the risk of hypertension and metabolic syndrome, both of which were ASCVD risk factors (11). A large Mendelian randomized study found that every 20 mmHg increase in systolic blood pressure could increase the risk of aortic stenosis by about three times (24). Metabolic syndrome may also contribute to aortic stenosis (25). Thus, hypertension and metabolic syndrome may be important comorbidities contributing to both ASCVD and VHD. In addition, inflammatory pathway may also be involved in the biological basis for this association. For example, women with later menarche had higher C-reactive protein (CRP) levels after menopause (26), while higher CRP levels were associated with greater risk of VHD (27). Moreover, women with later menarche were more likely to smoke and less likely to be well educated (17). A Norwegian study found that women with later menarche had shorter reproductive periods than women with earlier menarche (28). This suggested that women with later menarche had a shorter history of estrogen exposure and the protective effect of estrogen on cardiovascular health may be insufficient.

Although significant increase of prevalence in VHD was observed, the etiology of VHD has changed greatly as well in the past few decades. At present, the most important etiology of VHD was degeneration, followed by rheumatism (3, 29, 30). The prevalence of degenerative aortic stenosis increased significantly with age (1). Nazarzadeh et al. found that for every 10-year increase in age, the risk of degenerative VHD tripled (31, 32). Reproductive factors were emerging determinants of cardiovascular risk. Guidelines were gradually incorporating premature menopause as a cardiovascular risk factor (33). It may be necessary and reasonable to incorporate age at menarche when assessing VHD risk in women, especially for those with more preexisting risk factors. High-risk individuals with later menarche may need intensive interventions in terms of modifiable risk factors such as smoking and hypertension. However, how to use age at menarche to scientifically assess the risk of VHD was still challenging.

In conclusion, in this large inpatient cohort, later menarche was associated with higher risk of VHD. We should be aware of the higher VHD risk for women who have later onset of menarche.

## Limitation

Despite the large sample size and comprehensively assessed reproductive factors, our study has certain limitations that merit consideration. First, our study is based on inpatients and is not well representative of the general population. Second, this is an observational case–control study, and causality could not be determined. The age of menarche was dictated by the patient and cannot be objectively verified. The diagnosis of VHD is based on patient discharge diagnosis, and those with milder lesions may be missed. Therefore, the prevalence of VHD, especially non-rheumatic VHD, may be underestimated. Third, antihypertensive medications can influence the hemodynamics, which may bias VHD diagnosis, but there are no data regarding the temporal relations between use of antihypertension medication and VHD diagnosis. Fourth, 144,746 patients had missing data on VHD status and age at menarche, and we have compared these patients with the final sample (*n* = 105,707). The missing data do not appear to be random, which may lead to bias. However, we have a large sample size in final analysis, and most cardiometabolic factors are pretty much similar between excluded and included patients (Supplementary Table S1). Moreover, we further included preexisting individuals to verify the robustness of our results, and we found a largely similar trend (Supplementary Figure S1). Fifth, although we have adjusted the common cardiovascular risk factors in all the models, there may still be unknown confounding factors.

## Data Availability

The raw data supporting the conclusions of this article will be made available by the authors, without undue reservation.
