# Supplementary material for: Association of age at menarche with valvular heart disease: An analysis based on electronic health record (CREAT2109)
Source: Front Cardiovasc Med. 2023 Apr 17;10:1029456. doi: 10.3389/fcvm.2023.1029456 (PMC10149805; doi:10.3389/fcvm.2023.1029456)
Supplement: Supplementary file 1 [file Datasheet1.docx]

Supplementary Material

Association of age at menarche with valvular heart disease: An analysis based on electronic health record (CREAT2109)

**Zhiyu Sun, MD^1,2,3^, Yongjie Zhu, MSN^4^, Zhexun Lian, MD^1,2,*^, Mengqi Guo^1^, Xiaohong Lu, MSN^1^, Ting Song, MD^3^, Xiaoyan Sun, MD^3^, Luxin Feng, MD^3^, Yi Zhang, MD^5^, Yawei Xu, MD^5^, Hongwei Ji, MD^1,2,*^, Junjie Guo, MD^1,2,6,*^**

^1^Department of Cardiology, the Affiliated Hospital of Qingdao University, Qingdao, Shandong, China; ^2^Chinese patient-oriented metabolic and ischemic risk evaluation (CREAT) study, Qingdao, Shandong, China; ^3^Qingdao University, Qingdao Medical College, Qingdao, Peoples R China, ^4^Department of Emergency Medicine, the Affiliated Hospital of Qingdao University, Qingdao, Shandong, China; ^5^Department of Cardiology, Shanghai Tenth People’s Hospital, Tongji University, Shanghai, China; ^6^Qingdao Municipal Key Laboratory of Hypertension (Key Laboratory of Cardiovascular Medicine), Qingdao, Shandong, China;

*** Correspondence:** Hongwei Ji: [hongweijicn@gmail.com](mailto:hongweijicn@gmail.com); Zhexun Lian: [lianzx566@163.com](mailto:lianzx566@163.com); Guo Junjie: [guojunjie@qdu.edu.cn](mailto:guojunjie@qdu.edu.cn)

**Supplementary Figures and Table**


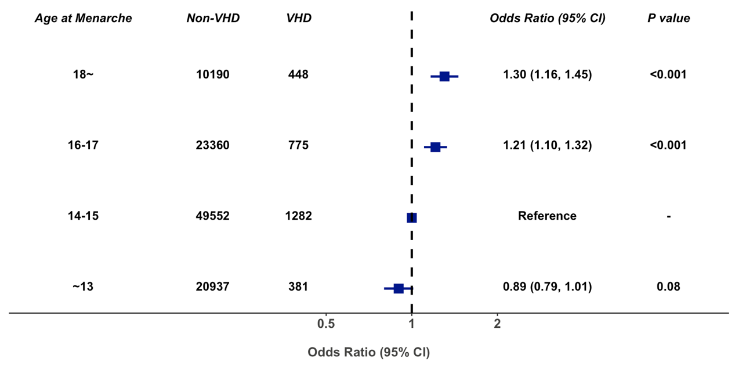


**Supplementary Figure S1: Sensitivity analysis including per-existing VHD patients.** VHD, valvular heart disease.

**Supplementary Table S1**

|  | ***Excluded*** | ***Included*** | ***P-value*** |
| --- | --- | --- | --- |
| ***N*** | 144746 | 105707 | - |
| Age, mean (SD), years | 50.71 (15.83) | 55.31 (13.62) | <0.001 |
| Smoking, n (%) | 2138 (1.6) | 1598 (1.5) | 0.023 |
| Systolic BP, mean (SD), mmHg | 123.16 (16.82) | 123.46 (18.09) | <0.001 |
| Anti-hypertensives, n (%) | 46452 (34.2) | 28829 (27.3) | <0.001 |
| DM, n (%) | 9031 (6.2) | 8689 (8.2) | <0.001 |
| BMI, mean (SD), kg/m^2^ | 24.57 (3.93) | 24.55 (3.65) | 0.216 |
| High density lipoprotein cholesterol, mean (SD), mmol/L | 1.44 (0.40) | 1.45 (0.37) | <0.001 |
| Low density lipoprotein cholesterol, mean (SD), mmol/L | 2.83 (0.90) | 2.86 (0.91) | <0.001 |
| Total cholesterol, mean (SD), mmol/L | 4.98 (1.31) | 4.62 (1.57) | <0.001 |
| Medical centers n (%) |  |  | <0.001 |
| East | 38371 (30.6) | 32574 (30.8) |  |
| North | 1656 (1.3) | 1640 (1.6) |  |
| South | 53161 (42.4) | 33732 (31.9) |  |
| West | 32070 (25.6) | 37761 (35.7) |  |
| Valvular heart disease, n (%) | 2096 (1.4) | 1668 (1.6) | 0.009 |
